# Microelectromechanical System-Based Electrochemical Seismometers with Two Pairs of Electrodes Integrated on One Chip

**DOI:** 10.3390/s19183953

**Published:** 2019-09-13

**Authors:** Xichen Zheng, Deyong Chen, Junbo Wang, Jian Chen, Chao Xu, Wenjie Qi, Bowen Liu

**Affiliations:** 1 School of Electronic, Electrical and Communication Engineering, University of Chinese Academy of Sciences, Beijing 100049, China; zhengxichen17@mails.ucas.ac.cn (X.Z.); jbwang@mail.ie.ac.cn (J.W.); chenjian@mail.ie.ac.cn (J.C.); xuchao16@mails.ucas.edu.cn (C.X.); qiwenjie16@mails.ucas.edu.cn (W.Q.); liubowen17@mails.ucas.edu.cn (B.L.); 2 State Key Laboratory of Transducer Technology, Institute of Electronics, Chinese Academy of Sciences, Beijing 100010, China

**Keywords:** microelectromechanical system, electrochemical seismometer, integrated sensing unit, high sensitivity, high fabrication repeatability

## Abstract

This paper presents microelectromechanical system (MEMS)-based electrochemical seismometers with two pairs of electrodes integrated on one chip. Both theoretical analysis and numerical simulations were conducted to reveal the working principle of the proposed electrochemical seismometers, finding that flow holes distributed on cathodes rather than anodes can produce significantly higher sensitivities. The proposed electrochemical seismometers were fabricated based on conventional micromachined processes with high fabrication repeatability. Sensitivity measurements of the proposed seismometers and their commercial counterpart of CME6011 were conducted, indicating the sensitivities of the proposed seismometer with flow holes on cathodes were two orders higher than the counterpart with flow holes on anodes and one order higher than CME6011 at dominant frequencies. Measurements of random ground motions based on the proposed seismometer with flow holes on cathodes and CME6011 were conducted, producing comparable noise levels without observable ground motions and high correlations in response to random events of ground motions. These results validated the functionality of the proposed electrochemical seismometers, which may contribute to seismic monitoring in the near future.

## 1. Introduction

A seismometer is a core element in seismic monitoring and geological prospecting [1,2,3]. According to the detecting mechanisms, seismometers can be divided into moving-coil seismometers, piezoelectric seismometers, electromagnetic seismometers, capacitive seismometers, optic-fiber seismometers, and electrochemical seismometers [4,5,6,7,8,9,10]. Table 1 lists the advantages and disadvantages of several kinds of mainstream seismometers. Among these, electrochemical seismometers are featured with no mechanical noises, high sensitivities, and large working dip. More importantly, no mass centering and lock is needed in electrochemical seismometers as the sensing unit is immersed in liquid, and thus, compared to the other sorts of seismometers, electrochemical seismometers are more suitable for ocean-bottom seismic monitoring in complex underwater environments [11].

The sensing units of traditional electrochemical seismometers were manufactured by conventional net-waving technologies, suffering from key problems of poor consistency, low yield, and high cost [12,13,14]. To address these issues, MEMS based sensing units of electrochemical seismometers were developed by both Chen et al. and Huang et al. [15,16]. Chen et al. proposed a sensing unit stacked up with layers of microfabricated electrodes and insulators to improve device consistency, which, however, required accurate alignments of different layers [15]. Subsequently, Huang et al. proposed a sensing unit with silicon nitride and platinum deposited on both sides of a silicon wafer in turn, realizing high-efficiency alignment. However, the device sensitivity was relatively low due to the limited electrode area, and the manufacturing process was time consuming because of focused ion beam etching [16]. To obtain high sensitivities, Deng et al. from our group developed a sensing unit with an anode and a cathode integrated on a single chip with much larger electrode areas [17]. Nevertheless, as a troublesome step, manual alignments were required in the assembly of sensing units, which were prone to chip damage. A new method based on parylene for manufacturing sensing units without manual alignments was then put forward, in which the device sensitivity was improved by decreasing the distances between electrodes to several micrometers by our group [18]. However, the parylene-based sensor was impractical as a result of poor adhesion between the parylene and the electrode layers.

To address the aforementioned problems, this paper presents new micromachined electrochemical seismometers with two pairs of sensing electrodes integrated on a single chip. In comparison to previously reported counterparts, the seismometers developed in this study are featured with high fabrication repeatability and high sensitivity because of enlarged electrode areas.

## 2. Device Structure and Working Principle

**Figure 1:**
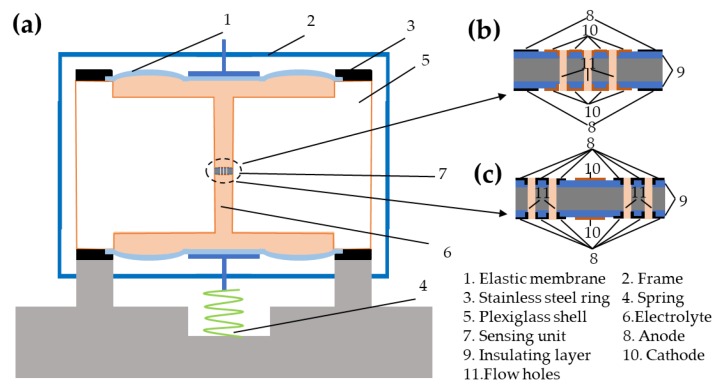
(**a**) Schematic of the MEMS based electrochemical seismometer, consisting of (1) two elastic membranes, (2) a frame, (3) two stainless steel rings, (4) a spring, (5) a plexiglass shell, (6) electrolyte, and (7) a sensing unit. The sensing unit is immersed in the electrolyte, which is encapsulated in the plexiglass shell sealed by two elastic membranes. The enlarged schematic of the sensing unit with (8) anodes, (9) insulting layers (10) cathodes, and (11) flow holes integrated on a single chip where flow holes are distributed on cathodes (**b**) or anodes (**c**), respectively.

As shown in Figure 1a, the MEMS-based electrochemical seismometers consist of two elastic membranes, a frame, two stainless steel rings, a spring, a plexiglass shell, electrolyte, and a sensing unit. Besides, the sensing unit is immersed in an electrolyte solution of tri-iodide and iodine ions (low-amount addition of iodine to potassium iodide solution), which is encapsulated in the plexiglass shell sealed by two elastic membranes. Furthermore, as shown in Figure 1b,c, the sensing unit includes two pairs of electrodes in symmetry, which are separated by insulating layers and integrated on one silicon chip. In addition, numerous through holes perpendicularly to the electrode surfaces function as flow ways, which are distributed on cathodes or anodes, respectively.

In this design, the cathodes were distributed on surfaces of the silicon wafer and the side walls of flow holes, whose electrode areas were more than 500 times larger than those of previous electrochemical seismometers based on single silicon wafers, where the areas of the cathodes were defined by side walls of flow holes [16]. Therefore, electrode areas of cathodes can be effectively enlarged, leading to significant improvements in device sensitivities. In addition, the inclusion of a pair of anode and cathode integrated on a plane could simplify the fabrication process without the steps of depositing extra insulating layers.

When a voltage is applied to the two electrodes on the same side of the sensing unit, there are reversible electrochemical reactions occurring around the anodes and the cathodes, which are $3I^{-}-2e^{-}\to I_{3}^{-}$ and $I_{3}^{-}+2e^{-}\to 3I^{-}$, respectively. When no vibration is applied to the seismometer, the concentration distribution of the ions on both sides is symmetrical, and thus the output voltage equates to zero. However, in case of vibrations, the ions flow relative to the sensing unit due to inertia, which results in an increase in concentration of tri-iodide ions around one cathode and a decrease in concentration of tri-iodide ions around the other cathode, generating a differential output current, with the amplitude and frequency indicating vibration status.

At first, owing to inertia, the velocity of external vibrations is converted to electrolyte velocity relative to electrodes, which then was converted to output voltage by electrochemical reaction. In fact, the electrolyte in seismometers can be considered as incompressible liquid in laminar flow. Therefore, we used Continuity equation and Navier–Stokes Equation to describe the velocity field:

(1) $$\rho \nabla \vec{V}=0$$

(2) $$\frac{\partial \vec{V}}{\partial t}=-\frac{\nabla \vec{P}}{\rho }+\frac{\mu }{\rho }\nabla^{2}\vec{V}+\vec{g}$$

where $\vec{V}$ is the velocity in the specified direction, $\vec{P}$ is the pressure, ρ is the density of the electrolyte, and $\vec{g}$ is the gravity acceleration. It is obvious the distribution of reaction ions will be affected by flow of the electrolyte, diffusion, and electromigration. Thus, the ion flux ($\vec{N}$) can be expressed by Nernst–Plank Equation as following:

(3) $$\vec{N}=-D\nabla C-\frac{zF}{RT}DC\nabla \varphi +C\vec{V}$$

The right side of the equation corresponds to flux changes caused by diffusion, electromigration, and convection, respectively. D is the diffusion coefficient, C is ion concentration, z is the charge of ions, F is the Faraday constant, R is the gas constant, T is the Calvin temperature, and φ is the potential. In addition, the electromigration component is very small as a result of the shielding effect of potassium ions, and the diffusion is the main component. At the same time, there is no flux at the boundary of channels. At the electrode boundary, the reactive ion flux satisfies the Bulter–Volmer Equation:

(4) $$2\vec{n}\cdot \vec{N_{I_{3}^{-}}}=-\frac{2\vec{n}\cdot \vec{N_{I^{-}}}}{3}=-2K_{a}C_{I_{3}^{-}}e^{-\frac{\alpha nF}{RT}*\left(U-E^{0}-\varphi \right)}+K_{c}C_{I^{-}}e^{\frac{\left(1-\alpha \right)nF}{RT}*\left(U-E^{0}-\varphi \right)}$$

in which $\vec{n}$ is a unit normal vector of electrode surface; K_a_ and K_c_ represent the rate constants of cathode and anode reactions, respectively; α is the charge transfer coefficient for the cathodic reaction; U is the imposed electrical potential between the electrodes; and $E^{0}$ is the equilibrium electrical potential. The relationship between output current on cathode and ion flux can be represented by the following Equation:

(5) $$I=\mathrm{nF}\overset{}{\underset{S}{\int }}\vec{n}\cdot \vec{N_{I_{3}^{-}}}ds$$

where n is the number of charge in single electrode reaction and equals to one, and S represents the area of cathode. The final output $I_{o}$ is given by:S

(6) $$U_{o}=(I_{1}-I_{2})*R$$

where $I_{1}$ and $I_{2}$ represents the output current of two cathodes, R represents voltage-current conversion resistance. Combining formulas mentioned above and based on known electrolyte velocity, $U_{o}$ can be calculated.

## 3. Simulation 

**Figure 2:**
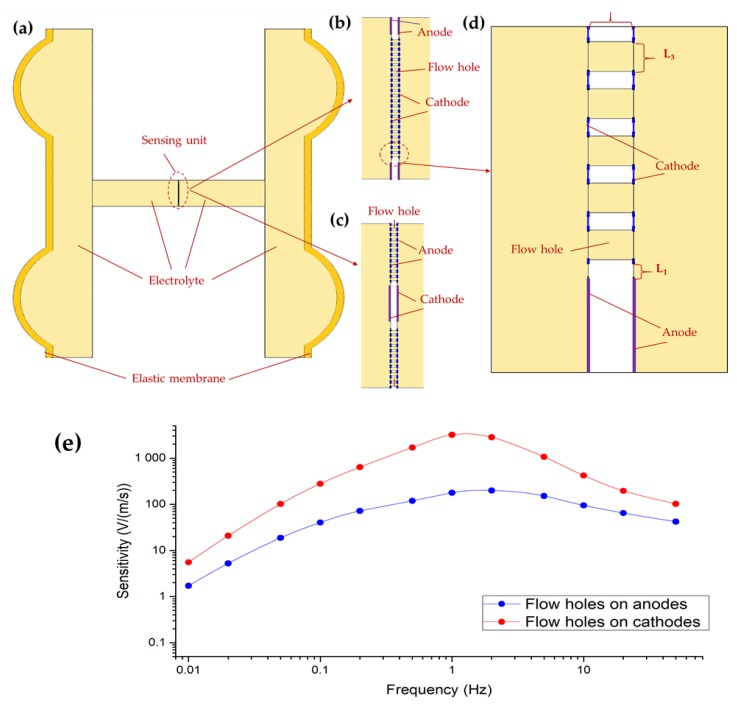
(**a**) The two-dimensional simulation model of the proposed MEMS-based electrochemical seismometers with two pairs of electrodes integrated on one chip in COMSOL Multiphysics, mainly consisting of the sensing unit, two elastic membranes, and the electrolyte. The simulation models of the sensing units with cathodes, anodes, and numerous flow holes distributed on cathodes (**b**) or anodes (**c**), respectively. (**d**) The enlarged simulation model of the sensing unit with flow holes on cathodes, where L_1_ represents the thickness of the insulating layer between the anode and the cathode, L_2_ represents both the thickness of the insulating layer between two pairs of anodes and cathodes and the length of the flow holes at the same time, and L_3_ represents the diameter of the flow holes. (**e**) Simulation results (sensitivity vs. input frequency of the volume force) of proposed seismometers with flow holes distributed on anodes or cathodes.

The electrochemical seismometers proposed in this study involved complex electrochemical analysis, and thus, finite element simulation (COMSOL Multiphysics 3.5, Stockholm, Sweden) was used for performance evaluation and optimization. As shown in Figure 2a, a two-dimensional model, consisting of the sensing unit with two pairs of electrodes integrated on one chip, two elastic membranes and electrolyte, coupled with (1) the physical field of solid mechanics, (2) the laminar flow field, and (3) the electric analysis field, was used to study the qualitative effect of key geometrical parameters of the sensing unit on the performance of the seismometers. Figure 2b,c shows the sensing units with two pairs of electrodes integrated on one chip, where the flow holes were distributed on cathodes or anodes with the same areas, respectively. Taking the model with flow holes distributed on cathodes as an example, the employed key geometrical parameters were as follows (see Figure 2d): the insulating space between anode and cathode (L_1_) is 100 μm; the insulating space between the two cathodes (L_2_) is 130 μm, which also represents the length of the flow holes; and the diameter of the flow holes (L_3_) is 100 μm. Initial concentrations of potassium iodide and iodine were set as 4000 mol/m^3^ and 40mol/m^3^, respectively. The inputting volume force (time piecewise function from 0.01 to 100 Hz) applied to the electrolyte was equivalent to the external vibration, which drove the liquid mass to flow.

In the simulation model of seismometers with two pairs of electrodes integrated on one chip, the electrolyte was considered as incompressible liquid in laminar flow, and the solid walls were in a no-slip boundary condition. Therefore, the physical fields of solid mechanics and laminar flow were described by the Continuity equation and the Navier–Stokes equation, respectively. At the same time, the electric analysis field was represented by Faraday’s law, the Bulter–Volmer condition and the Nernst–Plank equation under the condition of no flux at the channels’ boundary, where key parameters were given as follows: the charge transfer coefficient for the cathodic reaction is 0.5, the equilibrium electrical potential is 0.54 V, the diffusion coefficient of $I_{3}^{-}$ is 5.76 × 10^−10^ m^2^/s, and the diffusion coefficient of $I^{-}$ is 1.285 × 10^−9^ m^2^/s.

Figure 2e shows the simulation results of amplitude–frequency responses of proposed seismometers with two pairs of electrodes integrated on one chip in the frequency domain of 0.01–100 Hz, where the flow holes were distributed on anodes or cathodes, respectively. In addition, the voltage–current conversion resistor is set to 1 KΩ. The amplitudes for the electrochemical seismometers with holes on anodes vs. cathodes were quantified as 40.24 V/(m/s) vs. 278.47 V/(m/s) at 0.1 Hz, 177.58 V/(m/s) vs. 3173.02 V/(m/s) at 1 Hz, and 94.46 V/(m/s) vs. 419.84 V/(m/s) at 10 Hz, respectively. It was observed that the output amplitudes of the electrochemical seismometers increased firstly and then decreased with the increase of the frequency of the input volume force. This was because at the low-frequency domain the conversion efficiency between external vibrations and electrolyte movement was relatively low, while the rates of the electrochemical reactions on the electrodes were limited at the high-frequency domain. Furthermore, it was located that the output amplitudes of the seismometer with flow holes distributed on cathodes roughly one order higher than the counterpart with flow holes on anodes, owing to the fact that the concentration of tri-iodide ions around two cathodes was much higher than the counterparts with holes on anodes.

## 4. Fabrication 

**Figure 3:**
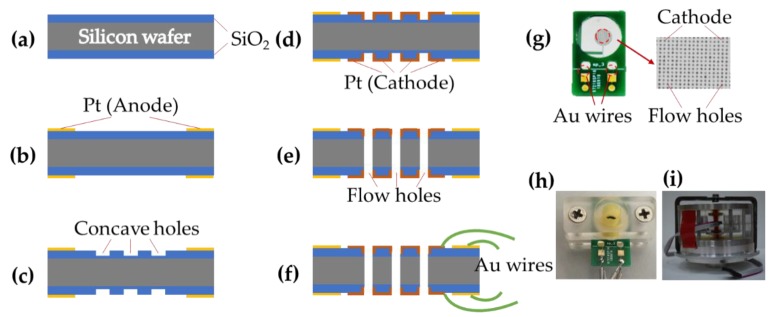
(**a**–**f**) The fabrication process of the sensing unit with two pairs of electrodes integrated on one chip, including key steps of (**a**) deposition of SiO_2_ layers, (**b**) deposition and patterning of anodes, (**c**) formation of concave holes in SiO_2_ layers, (**d**) deposition and patterning of cathodes, (**e**) formation of flow holes on cathodes, and (**f**) connection with Au wires. Pictures of (**g**) a fabricated sensing unit, (**h**) the assembled sensing unit, and (**i**) the assembled electrochemical seismometer.

The proposed microelectromechanical system-based electrochemical seismometers with two pairs of electrodes integrated on one chip were based on conventional microfabrication including key steps of metal deposition, photolithography, and deep reactive ion etching (DRIE) (see Figure 3). 

(a) SiO_2_ layers (1 μm) for insulating were deposited on both sides of the wafer by wet oxidation after wafers were placed in boiled sulfuric acid and deionized water for thorough cleaning.

(b) At first, the pattern of the photoresist mask (AZ1500 photoresist) was prepared on one side of the substrate by photolithography, followed by the cleaning step based on oxygen plasma (3 min). Ti (40 nm) and platinum Pt (200 nm) were sputtered in sequence on the patterned surface of the SiO_2_ film after oxygen plasma (30 s). Subsequently, the anode was formed by lift-off technology where acetone, alcohol, and deionized water were used in turn to remove the underneath photoresist. Finally, the other side of the substrate was processed in the same way, producing symmetrical anodes on both sides of the silicon wafer.

(c) For protecting the surface of anode, the photoresist mask pattern (AZ4620 photoresist) was formed on one side of the substrate by photolithography. Then, about 800 nm of the SiO_2_ layer was removed by trifluoromethane etching to form flow holes. Afterwards, the photoresist mask was removed. In the end, the other side was fabricated with the same steps where the concave holes were distributed symmetrically on both SiO_2_ layers.

(d) Firstly, the pattern of cathodes was transferred from the mask to the surface of SiO_2_ layers by photolithography (AZ1500 photoresist). Then Ti (40 nm) and Pt (200 nm) were sputtered successively on the surface of the SiO_2_ film. Subsequently, the cathodes were formed by lift-off technology. Finally, the other side of the substrate was processed in the same way, generating symmetrical cathodes on both sides of the silicon wafer.

(e) The pattern of the photoresist mask (AZ4620 photoresist) was formed on one side of the silicon wafer. Next, the remaining SiO_2_ layer was etched by trifluoromethane from the front side. Then, the silicon layer was removed completely by DRIE. Finally, the SiO_2_ layer on the other side was removed by trifluoromethane etching and then the patterned wafer was cleaned up.

(f) The patterned four electrodes were connected to four pads on two printed circuit boards respectively by wire bonding leveraging Au wires.

Figure 3g shows a fabricated sensing unit, where the dimensions of the sensing unit before and after wire bonding were 12 mm by 15 mm and 12 mm by 20 mm, respectively. Figure 3h shows an assembled sensing unit, where an O-ring and an organic glass elements were placed on each side of a fabricated sensing unit, and the screws at both ends of the organic glass were tightened. In addition, a picture of a fabricated cathode with hundreds of flow holes (diameters of 100 μm) was also included in Figure 3g. Figure 3h shows the prototype of an assembled electrochemical seismometer, including key components of two elastic membranes, a plexiglass shell, and an assembled sensing unit, which were sealed together by mechanical compression.

In this study, all the steps mentioned above belong to the mature silicon-based fabrication technology. In addition, both the silicon dioxide layers fabricated by wet oxidation and the electrode layers manufactured by sputtering on the silica layer are stable enough. Thus, the seismometers developed in this study are featured with high fabrication repeatability

## 5. Devices Characterizations 

In order to compare the performances of the developed seismometers, a commercially available electrochemical seismometer CME6011 was used as a reference. Moreover, comparisons of the proposed seismometers with holes distributed on anodes or cathodes were conducted to verify the simulation results.

### 5.1. Amplitude–Frequency Response

**Figure 4:**
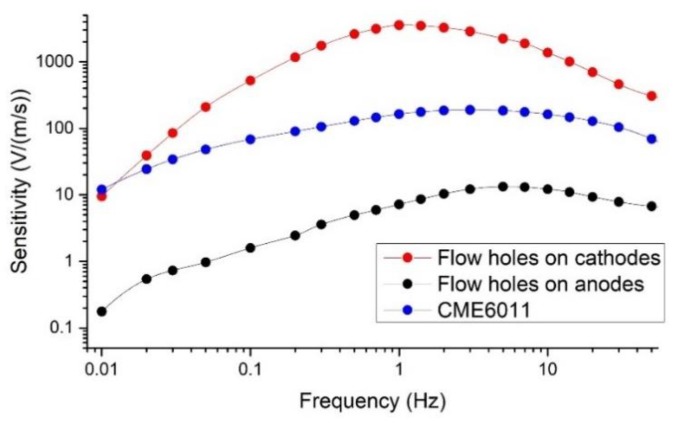
The sensitivities of the proposed electrochemical seismometers with holes on cathodes or anodes and CME6011 as a function of the frequency of the volume force.

The experiments to characterize amplitude-frequency responses of electrochemical seismometers were conducted on an ultra-low-frequency vibration table (National Institute of Metrology), where the sinusoidal voltage generated by a waveform generator and amplified by a power amplifier was converted to the input velocity. In addition, the voltage-current conversion resistors were set to 1 KΩ. Figure 4 shows the sensitivities of the proposed seismometers with holes on cathodes or anodes and CME6011, which were quantified as 521.28 V/(m/s) vs. 1.59 V/(m/s) vs. 68.05 V/(m/s) at 0.1 Hz, 3555.57 V/(m/s) vs. 7.16 V/(m/s) vs. 163.13 V/(m/s) at 1 Hz, and 1369.22 V/(m/s) vs. 12.14 V/(m/s) vs. 162.03 V/(m/s) at 10 Hz, respectively. Based on the experimental results, the sensitivities of the proposed seismometer with flow holes on cathodes were more than two orders higher than the counterpart with flow holes on anodes at 1 Hz, which was one order higher than the simulation results. However, the trend of amplitude–frequency responses of the proposed seismometers with flow holes on cathodes or the anodes was consistent with the simulation results. That was because two-dimensional simulations can only provide a qualitative rather than a quantitative estimation of device performances.

In addition, the sensitivities of the proposed seismometer with flow holes on cathodes were significantly higher than the counterpart with flow holes on anodes as a result of faster reduction reaction on cathodes. When the seismometer was subjected to vibration, the electrolyte solution would move in the direction of the flow holes. Therefore, for seismometers with flow holes distributed on the anode, most of the products (tri-iodide ions) of the anodic reaction would be taken away by the solution through the flow holes and seldom flowed to the cathode. As for seismometers with flow holes distributed on the cathode, most of the products of the anodic reaction would flow to the flow holes and the cathodes. At the same time, tri-iodide ions in the cathodic reaction are mainly the products of the anodic reaction due to low concentration of tri-iodide ions in electrolyte. Therefore, the concentration of tri-iodide ions around two cathodes was much higher than the counterparts with holes on anodes, leading to faster reduction reaction and higher sensitivities.

As shown in Figure 4, in comparison to CME6011, the sensitivities of the proposed electrochemical seismometer with flow holes on cathodes were comparable at the low-frequency domain, more than one order higher at the intermediate-frequency domain and about five times higher at the high-frequency domain. That was because of enlarged electrode areas and decreased thickness of insulating layers, resulting in an increase of differential tri-iodide ions flux. These results validated the functionality of the proposed electrochemical seismometers in this study. However, the 3dB working bandwidth (the frequency range where the amplitude is higher than 0.707 times of the maximum) of the proposed electrochemical seismometer with flow holes on cathodes was narrower than that of CME6011 and that of the proposed electrochemical seismometer with flow holes on anodes. Thus, it is necessary to balance between the sensitivities and the 3dB working bandwidth in various applications.

### 5.2. Self-Noise Level

**Figure 5:**
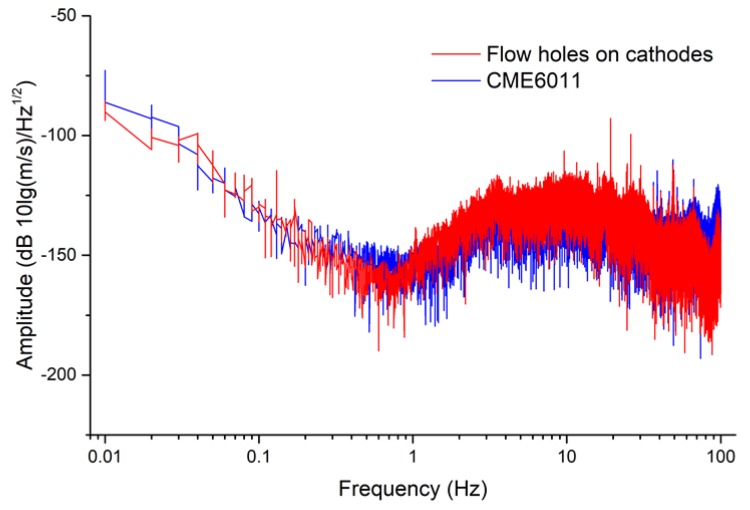
Self-noise spectrum densities of both the proposed electrochemical seismometer with flow holes on cathodes and CME6011.

In order to further characterize the self-noise level of the proposed electrochemical seismometer with flow holes on cathodes, it was placed side by side with CME6011 to collect self-noise data. Figure 5 shows the self-noise spectrum densities of both the proposed electrochemical seismometer with flow holes on cathodes and CME6011. Based on these results shown in Figure 5, the noise spectrum densities of the proposed seismometer and CME6011 were quantified as −139.75 dB (10l g(m/s)/Hz1/2) vs. −136.33 dB at 0.1 Hz, −161.28 dB vs. −159.85 dB at 1 Hz, and −134.96 dB vs. −127.35 dB at 10 Hz, respectively. Comparable noise levels of the proposed electrochemical seismometer with flow holes on cathodes and CME6011 were located, validating the functionality of the proposed electrochemical seismometer in this study.

### 5.3. Transient Response 

**Figure 6:**
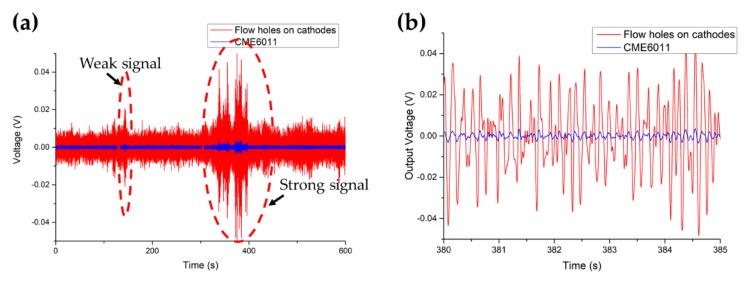
(**a**) The time-domain responses of both the proposed electrochemical seismometer with flow holes on cathodes and CME6011 with two events of ground motions, including a weak signal (138–144 s) and a strong signal (330–400 s) with details of recorded signals from 380 s to 385 s shown in (**b**).

Figure 6a shows the transient responses of both the proposed electrochemical seismometer and CME6011 with two events of ground motions, including a weak signal (138–144 s) and a strong signal (330–400 s). Figure 6b shows the details of recorded strong signals from 380 s to 385 s, where the frequency of output voltage was about 7 Hz. Based on these results shown in Figure 6b, the output voltage amplitudes of these two types of seismometers were quantified as 0.034 V vs. 0.0031 V (381.72 s) and 0.046 V vs. 0.0039 V (384.55 s), demonstrating that the transient responses of the proposed seismometers with holes on cathodes were about 10.97 times and 11.79 times those of CME6011, which was consistent with the test results in amplitude–frequency response. In addition, the correlation coefficient of these two types of electrochemical seismometers was quantified as 0.979 after normalization of output voltage, further validating the functionality of the proposed electrochemical seismometer in this study.

## 6. Conclusion

This paper demonstrated MEMS based electrochemical seismometers with two pairs of electrodes integrated on a chip. Both simulation and experimental results showed that the sensitivities of the proposed seismometer with flow holes on cathodes were one to two orders higher than the counterpart with flow holes on anodes. In addition, compared to CME6011, the proposed seismometer with flow holes on cathodes indicated significantly higher sensitivities and comparable noise levels. These results validated the functionality of the proposed electrochemical seismometers, which may contribute to seismic monitoring in the near future.

## Figures and Tables

**Table 1:** The comparison of several kinds of mainstream seismometers.

Types	Advantages	Disadvantages
Moving-Coil	Passive, Low cost, Simple structure	Poor low-frequency performance
Optic-Fiber	High sensitivity	High cost, High mechanical noise
Capacitive	Broadband, wide dynamic range	Fragile, Small working dip
Electrochemical	No mechanical noises, High sensitivity, large wrking dip	Complex principle
